# Acne vulgaris: prevalence, severity, and impact on quality of life and self-esteem among Egyptian adolescents

**DOI:** 10.1186/s42506-020-00056-9

**Published:** 2020-11-05

**Authors:** Kholoud Tayel, Medhat Attia, Naglaa Agamia, Noha Fadl

**Affiliations:** 1 Adolescent and School Health, Department of Family Health, High Institute of Public Health, Alexandria University, 165 El-Horrya Avenue, Alexandria, Egypt; 2 Mental Health, Department of Family Health, High Institute of Public Health, Alexandria University, 165 El-Horrya Avenue, Alexandria, Egypt; 3 Department of Dermatology, Venereology and Andrology, Faculty of Medicine, Alexandria University, Champollion Street, El-Khartoum Square, Azarita Medical Campus, Alexandria, Egypt

**Keywords:** Acne, Quality of life, Self-esteem, Adolescents, Egypt

## Abstract

**Background:**

Acne vulgaris is the most common dermatoses affecting adolescents with significant impact on their quality of life (QoL). The current study aimed to estimate the prevalence of acne, severity, and its impact on QoL and self-esteem among Egyptian adolescents.

**Methods:**

A cross-sectional study was conducted. A total of 787 students were selected using multistage stratified random sampling from 12 secondary schools in Alexandria, Egypt. Data was collected using a self-reported questionnaire, and clinical examination was performed. Severity of acne and its impact on QoL and self-esteem were assessed using the Global Acne Grading System (GAGS), Cardiff Acne Disability Index (CADI), and Coopersmith self-esteem scale, respectively.

**Results:**

Prevalence of self-reported acne was 34.7%. Females significantly reported acne more frequently than males (39.1% vs. 30.3%, *p* = 0.009). Prevalence of clinically confirmed acne was 24.4%, with higher rates among females (28.6%) than males (20.2%, *p* = 0.006). The majority of students had mild acne (75.5%). CADI showed that 11.4% had severe disability. A significant medium positive correlation between GAGS and CADI was found (*r* = 0.338, *p* < 0.01). Among acne group, low self-esteem was more prevalent among females (67.0%) than males (45.0%, *p* = 0.004).

**Conclusions:**

Acne is a common problem among Egyptian school-aged adolescents with higher prevalence and impact in females. Our findings should alert health professionals and school authorities to timely identify, manage, and educate adolescents with acne.

## Introduction

The skin is the body’s main interface with the external world [1]. It is considered as the body’s major public relations tool. Skin disorders have a negative impact on individuals, especially in acceptance of their own image and in quality of life (QoL) [2]. The prevalence of acne varies from 28.9 to 91.3% among adolescents [3]. Acne is a chronic inflammatory disease of pilosebaceous unit characterized by seborrhea, comedones, papules, pustules, nodules, cysts, and in some cases scars and keloids that persist for the rest of life [4].

Acne is often visible on the face heightening issues of body image and socialization. Therefore, it is not surprising that an individual with facial acne may develop significant psychosocial disability [5]. Adolescents are constantly concerned with images of perfection. When the exceedingly visible burden of acne is added, it might be the proverbial straw that tips them over into some functional or emotional abyss [6]. Studies on the psychological impact of acne have documented dissatisfaction with appearance, embarrassment, self-consciousness, and low self-esteem [7]. In this age group, patients are not mature enough to face such impact caused by acne deforming lesions. Considering all negative repercussions in a psychosocial context, acne has a great potential for jeopardizing QoL [8].

There is a need for studies in which the impact of acne on adolescents’ quality of life is investigated by reliable measures. Such investigations might attract the attention of dermatologists to the psychosocial aspects of acne. Even this may have a contribution as a first step for adolescents to seek help for acne [9]. The aims of the current study, were to estimate the prevalence and severity of acne and to determine its impact on QoL and self-esteem among adolescents in Alexandria, Egypt. We hypothesized that adolescents with acne would experience higher disability of QoL and lower self-esteem.

## Methods

### Study design, setting, and sampling method

A cross-sectional study was conducted using multistage stratified random sampling. Students were selected from twelve secondary schools (six private and six public) in Alexandria, between February and May 2016. Three educational districts (the most crowded) were chosen from the eight educational districts in Alexandria Governorate. From each of the selected educational district, two public schools and two private schools (i.e., one school for girls and one school for boys) were chosen at random. The sample of the students was determined according to proportional allocation method based on the total number of students in the selected educational districts. The total number of students in the most crowded three educational districts (East, West, and Montazah) was 82,599. East, West, and Monatzah educational districts included 29,539, 28,903, and 24,157 students, respectively. Using proportional allocation method, 275, 270, and 225 students were selected from East, West, and Montazah educational districts, respectively. Nearly equal number from each of the selected schools was chosen. One class from the 1st and 2nd secondary grades was chosen at random. All students in the selected classes were included in the study (with a total of 24 classes; 411 from 1st secondary and 376 from 2nd secondary grades).

The sample size was calculated using EPI-Info 2002 software, and based on the acne prevalence of 54.2% among adolescents from a previous study [10], the minimal required sample size was 770 students using 5% accepted degree of precision, α of 0.05, and power of 80%.

### Data collection

Schools were visited by the researcher, and data was collected using a self-reported pre-designed structured questionnaire. Each student was examined for the presence of acne, and they had their acne severity graded. The examination for acne included the face only. Severity of acne and its impact on QoL and self-esteem were assessed using Global Acne Grading System (GAGS), Cardiff Acne Disability Index (CADI), and Coopersmith self-esteem scale, respectively:

1. *Global Acne Grading System* (GAGS) was used to grade facial acne. This grading system calculates the severity of acne through the combined assessment of the types of acne lesions (comedones, papules, pustules, and nodules) and their anatomic location (forehead, cheeks, nose, and chin). The GAGS considers five locations on the face, with a factor at each location based roughly on surface area, distribution, and density of pilosebaceous units. Each type of acne lesion is given a value depending on severity: no lesions = 0, comedones = 1, papules = 2, pustules = 3, and nodules = 4 [11].
  Each of the location was graded separately on 0–4 scale, with the most severe lesion within that location determining the local score. The severity was then graded according to the global score which is the summation of all local scores. A score of 1–6 was considered mild; 7–18, moderate; 19–26, severe; and 27–32, very severe. The maximum score was 32 [11].
2. *The Arabic version of Cardiff Acne Disability Index* (CADI) is a well-validated self-reported questionnaire designed for measuring disability induced by acne in teenagers and young adults [12].

The Cardiff Acne Disability Index consists of five questions with a Likert scale, four response categories (0–3). The five questions relate to feeling of aggression, frustration, interference with social life, avoidance of public changing facilities and appearance of the skin—all over the last month—and an indication of how bad the acne was now. The CADI score was calculated by summing the score of each question resulting in a possible maximum of 15 and minimum of 0. CADI scores were graded as low (0–4), medium (5–9), and high (10–15). The lower the cumulative CADI score, the lower the level of disability experienced by the student while a higher score indicated a higher level of disability [12]. The internal consistency reliability of the CADI was found to be high, Cronbach’s alpha coefficient = 0.962.

3. *The Arabic version of the Coopersmith Self-Esteem inventory* is a self-report rating scale to assess self-esteem in children. It consists of 25 items. Each item consists of 2 choices with a score of 0–1, and the final score ranges from 0 to 25. Those with total score ≥ 20 were considered as high self-esteem, ≥ 14 were considered as low self-esteem, and > 14 to < 20 were considered as average self-esteem [13]. Cronbach’s alpha value for the internal consistency reliability of the scale was 0.743.

### Statistical analysis

Collected data was reviewed for completeness and accuracy, coded, computed, cleaned, and analyzed using Statistical Package for the Social Sciences (SPSS version 21.0). Descriptive statistics were performed to describe the students’ characteristics. Chi-square test (*χ*^2^) was used to test the significance of qualitative variables. The Monte Carlo test was used when chi-square significance test (*χ*^2^) was not applicable. Pearson’s correlation coefficient (*r*) was used to measure the strength of the relationship between CADI and GAGS. Results were considered significant at *p* value ≤ 0.05.

## Results

A total of 787 students were recruited in the present study, comprising 396 males (50.32%) and 391 females (49.68%). Their age ranged from 15 to 18 years with a mean of 16.31 ± 0.725 years. Regarding parents’ education, 62.01% and 65.44% of the students’ fathers and mothers were university graduates, respectively. Public school students were 409 (51.97%); meanwhile, 378 students were in private schools (48.03%).

The prevalence of self-reported acne and clinically confirmed acne was 34.69% and 24.39%, respectively. Regarding gender difference, females reported acne more frequently than males did (39.13% vs. 30.30, *p* = 0.009). Similarly, prevalence of clinically confirmed acne was higher among females (28.64%) than males (20.20%, *p* = 0.006) (Table 1). It was found that a significantly higher proportion of students (112 students) in private schools had clinically confirmed acne compared to those (80 students) in public schools (29.63% vs. 19.56%, *p* = 0.001). Regarding health-seeking behavior, 128 students with clinically confirmed acne (66.67%) reported visiting a health facility or pharmacy concerning their acne. Doctors were more consulted than pharmacists (74.22% vs. 25.78%). Forty-eight students (37.50%) sought medical advice within 3 months after developing acne, while 36 students (28.12%) waited for more than 1 year until seeking medical advice. Facial cleansers were the most frequently self-administered treatments (59.42%) followed by facial masks (31.71%) and medicated soap (23.44%).

**Table 1:** Acne among secondary school-aged adolescents, Alexandria, Egypt, 2016

	Overall, ***n*** (%)	Male, ***n*** (%)	Female, ***n*** (%)	***p*** value
**Self-reported acne (*n*** **= 787)**				0.009*
● Yes	273 (34.69)	120 (30.30)	153 (39.13)	
● No	514 (65.31)	276 (69.69)	238 (60.87)	
**Clinically confirmed acne (*n*** **= 787)**				0.006*
● Yes	192 (24.39)	80 (20.20)	112 (28.64)	
● No	595 (75.60)	316 (79.80)	279 (71.36)	
**Acne severity (*n*** **= 192)**				0.586
● Mild	145 (75.52)	58 (72.50)	87 (77.68)	
● Moderate	44 (22.92)	20 (25.00)	24 (21.43)	
● Severe	3 (1.56)	2 (2.50)	1 (0.89)	
**Disability of QoL (CADI) (*n*** **= 192)**				0.103
● Mild	94 (48.96)	43 (53.75)	51 (45.54)	
● Moderate	76 (39.58)	25 (31.25)	51 (45.54)	
● Severe	22 (11.46)	12 (15.00)	10 (8.92)	
**Mean ± SD** 4.95 ± 3.21				
**Self-esteem among acne group (*n*** **= 192)**				0.004*
● Low	111 (57.81)	36 (45.00)	75 (66.96)	
● Average	70 (36.46)	36 (45.00)	34 (30.36)	
● High	11 (5.73)	8 (10.00)	3 (2.68)	

*p < 0.05 (significant)

Using GAGS, 75.52% of students with clinically confirmed acne had mild acne, 22.92% had moderate acne, and only 1.56% had severe acne. Gender difference in terms of acne severity was not statistically significant; however, moderate and severe forms of acne were more common among males than females (*p* = 0.586) (Table 1).

The CADI score showed that 48.96% of the students with clinically confirmed acne experienced mild level of disability while 11.46% of the students with acne had severe level of disability. No association between QoL disability and gender was found (*p* = 0.103) (Table 1). Among students with clinically confirmed acne, low self-esteem was significantly more prevalent among females (66.96%) than males (45.00%, *p* = 0.004) (Table 1).

Specific responses of CADI are demonstrated in Table 2. It was reported that 28.12% of students with clinically confirmed acne felt aggressive, frustrated, or embarrassed either a lot or very much as a result of having acne; 17.18% stated that acne caused moderate and severe social interference affecting their activities; and 14.59% of them suffered from avoidance of public changing most of the time and all the time. Regarding impact of acne on their psychological state, 28.12% were usually concerned about the disease and 14.58% were very depressed and miserable. Furthermore, nearly one fourth (24.48%) of students perceived acne as a major problem and 11.46% mentioned that acne was the worst it could possibly be (Table 2).

**Table 2:** Specific responses to Cardiff Acne Disability Index (CADI)

	***n*** (%) (***n*** = 192)
**Felt aggressive, frustrated, or embarrassed**	
● Not at all	68 (35.42)
● A little	70 (36.46)
● A lot	32 (16.66)
● Very much indeed	22 (11.46)
**Mean ± SD** (1.04 ± 0.99)	
**Social interference**	
● Not at all	100 (52.08)
● Occasionally or in only some activities	59 (30.73)
● Moderately, in most activities	19 (9.90)
● Severely, affecting all activities	14 (7.29)
**Mean ± SD** (0.72 ± 0.91)	
**Avoidance of public changing**	
● Not at all	137 (71.35)
● Occasionally	27 (14.06)
● Most of the time	21 (10.94)
● All of the time	7 (3.65)
**Mean ± SD** (0.47 ± 0.83)	
**Patient psychological state**	
● Not bothered	42 (21.88)
● Occasionally concerned	68 (35.42)
● Usually concerned	54 (28.12)
● Very depressed and miserable	28 (14.58)
**Mean ± SD** (1.35 ± 0.98)	
**Subjective assessment of acne severity (perceived as a problem)**	
● Not a problem	21 (10.94)
● A minor problem	102 (53.12)
● A major problem	47 (24.48)
● The worst it could possibly be	22 (11.46)
**Mean ± SD** (1.36 ± 0.82)	

**Fig. 1:**
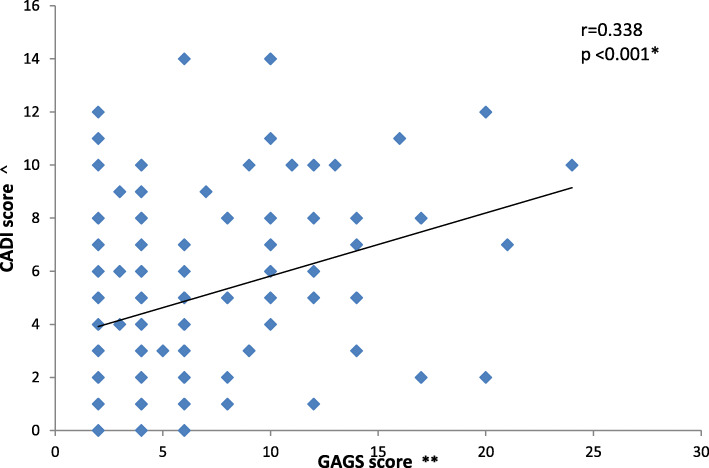
Pearson’s coefficient demonstrating correlation between acne severity (Global Acne Grading Score**) and degree of disability of quality of life (CADI score^). **Global Acne Grading System. **^**Cardiff Acne Disability Index

More disability was experienced by students having more severe disease (Table 3). Figure 1 reveals a significant medium positive correlation between acne severity and degree of QoL disability (*r* = 0.338, *p* < 0.001).

**Table 3:** Severity of acne and quality of life disability in secondary school adolescents, Alexandria, Egypt, 2016

Severity of acne (***n*** = 192)	QoL disability			***p*** value
	Mild, ***n*** (%)	Moderate, ***n*** (%)	Severe, ***n*** (%)	
● Mild	83 (57.24)	52 (35.86)	10 (6.90)	< 0.001* (Monte Carlo)
● Moderate	11 (25.00)	23 (52.27)	10 (22.73)	
● Severe	0 (0.00)	1 (33.33)	2 (66.67)	

*p < 0.05 (significant)

## Discussion

The current study showed that nearly one fourth of the sampled students were affected by acne and it was found to have negative implications on QoL, correlating with severity. More or less similar findings were reported among Egyptian school-aged adolescents by Hassan et al. and El-Hamd et al. (21.6% and 33.5%, respectively) [14, 15]. However, another Egyptian study showed a higher prevalence among secondary school students (54.2%) [10]. Higher prevalence of acne was also reported in China (38%) [4], Nigeria (64.4%) [16], Kuwait (67.1%) [17], Lithuania (82.9%) [18], and Brazil (96%) [19]. Comparison of prevalence rates between studies is sometimes difficult due to differences in study design, study settings, participants’ characteristics, seasonal variations, genetics, and diet; however, our finding revealed that acne is a common problem among Egyptian adolescents.

Although both genders were nearly equally represented in the current study, clinically confirmed acne was significantly higher among females than males. Female predominance was observed by AlKhabbaz et al. [17], Campbell et al. [20], and Noorbala et al. [21]. This may be due to hormonal changes during menstruation or higher level of stress among females. Additionally, it was found that the prevalence of self-reported acne among the students was higher than the prevalence of clinically confirmed acne. This was reported among adolescents in Turkey and Norway [9, 22] as they commonly tend to over-report their acne. Regarding gender difference, the overall prevalence of self-reported acne was significantly higher among girls than boys. The gender distribution was in accordance with that found among Lithuanian and Japanese adolescents [18, 23]. This might be due to a higher appearance consciousness of females.

The present study showed that prevalence of clinically confirmed acne was significantly higher among private school students than public school students. Okoro et al. showed a similar finding [16]. It is generally thought that students in private schools are likely from higher socioeconomic background. Socioeconomic status is an important factor in determining the life style, body mass index, and dietary habits that may influence the occurrence of acne among adolescents.

The majority of students, in the current study, had mild acne. Mild acne was the predominant grade of acne among adolescents in Nigeria (88.6%) and India (81.9%) [16, 24]. On the contrary, more moderate/severe grades of acne (67%) compared to mild grades (33%) were found in a Turkish hospital-based study [25]. This is not surprising, as persons with more severe diseases are likely to seek medical intervention; thus, hospital-based studies are likely to yield more severe forms of acne compared to community-based ones such as the present study.

The current study emphasized that acne is associated with significant morbidity and decrease in QoL among adolescents. A considerable percentage of students had high CADI scores (11.4%), indicating a high psychosocial burden from acne. The level of psychosocial impairment that can result from acne may be compared to that associated with epilepsy, asthma, diabetes, back pain, arthritis, and coronary heart disease [26].

The mean score of CADI, in the present study, was higher than that reported among Serbian [27], Nigerian [28], and Iranian [21] adolescents; the recorded mean score of CADI was 2.87, 3.4, and 3.8, respectively. On the other hand, a higher score of 6.22 was obtained in a hospital-based study by Pandey et al. [29]. These discrepancies among studies are likely a result of the difference in acne severity, cultural differences, and individuals’ coping ability.

Reviewing the responses to individual questions is important when evaluating QoL measures. Subjective assessment of severity of acne, psychological state, and feeling of aggression was found to have higher impact on QoL. Comparing with Malaysian and Nigerian adolescents, these questions also elicited the highest percentage of responses [2, 28], implying that these questions allowed the students to express the actual impact of acne on their QoL. Avoidance of public changing facilities or wearing swimming costume activities yielded the least impact on QoL. This was the observation a Serbian study [27]. This may imply either that extra facial acne is uncommon or that the presence of such lesions does not affect the students’ utilization of public changing facilities or dressing mode, or even that the question is irrelevant to the majority of students.

The present study showed that the negative impact on QoL increased with acne severity. It also demonstrated a significant medium positive correlation between the total score of CADI and acne severity. Desai et al. [30], El-Hamd et al. [15], and Hosthota et al. [31] also reported a positive correlation between severity of acne and CADI among school-aged adolescents. This implies that impact of acne on QoL must be considered in the management of facial acne.

Among those with clinically confirmed acne, self-esteem was significantly worse for females than males. The majority of researchers found that females with acne were more likely to have greater self-consciousness and self-perceived stress, lower self-esteem and feelings of self-worth, poorer body satisfaction, lower self-attitude, and greater feelings of uselessness [32].

### Study limitations

Findings from the current study should be interpreted in light of the following limitations. The cross-sectional design of the present study limits the possibility of establishing temporality. Additionally, the psychosocial status was assessed through self-reporting. A systematic psychiatric diagnostic through interview of the patients would have given a better assessment of the condition.

## Conclusions

Despite these limitations, the present study highlighted the problem of acne among Egyptian adolescents. It revealed that acne has a substantial impact on QoL among adolescents. The more the severity of the disease, the higher the disability of QoL. Timely diagnosis and treatment of acne, in combination with the appropriate mental health support, can be an important factor in reducing psychosocial stress among adolescents. The treatment of acne should be guided by both acne severity and degree of QoL impairment. Routine use of QoL assessment tools in the care of patients with acne represents an opportunity for the physicians to better incorporate the values and concerns of the patient into their care. A strong physician-patient relationship and thorough history taking may help to identify adolescents at risk for the adverse psychosocial effects of acne. Additionally, there is a need for accessible, accurate health education programs for adolescents and school students to pave the way to appropriate management of acne.

## Data Availability

Data will be available on reasonable request. Please contact the corresponding author for data requests.

## Abbreviations

QoL: Quality of life
GAGS: Global Acne Grading System
CADI: Cardiff Acne Disability Index
